# Mechanism of nuclear factor of activated T-cells mediated FasL expression in corticosterone -treated mouse Leydig tumor cells

**DOI:** 10.1186/1471-2121-9-31

**Published:** 2008-06-12

**Authors:** Wei-Ran Chai, Yong Chen, Qian Wang, Hui-Bao Gao

**Affiliations:** 1 Department of Biochemistry and Molecular Biology, Shanghai JiaoTong University School of Medicine, Shanghai 200025, PR China

## Abstract

**Background:**

Fas and FasL is important mediators of apoptosis. We have previously reported that the stress levels of corticosterone (CORT, glucocorticoid in rat) increase expression of Fas/FasL and activate Fas/FasL signal pathway in rat Leydig cells, which consequently leads to apoptosis. Moreover, our another study showed that nuclear factor of activated T-cells (NFAT) may play a potential role in up-regulation of FasL during CORT-treated rat Leydig cell. It is not clear yet how NFAT is involved in CORT-induced up-regulation of FasL. The aim of the present study is to investigate the molecular mechanisms of NFAT-mediated FasL expression in CORT-treated Leydig cells.

**Results:**

Western blot analysis showed that NFAT2 expression is present in mouse Leydig tumor cell (mLTC-1). CORT-induced increase in FasL expression in mLTC-1 was ascertained by Western Blot analysis and CORT-induced increase in apoptotic frequency of mLTC-1 cells was detected by FACS with annexin-V labeling. Confocal imaging of NFAT2-GFP in mLTC-1 showed that high level of CORT stimulated NFAT translocation from the cytoplasm to the nucleus. RNA interference-mediated knockdown of NFAT2 significantly attenuated CORT-induced up-regulation of FasL expression in mLTC. These results corroborated our previous finding that NFAT2 is involved in CORT-induced FasL expression in rat Leydig cells and showed that mLTC-1 is a suitable model for investigating the mechanism of CORT-induced FasL expression. The analysis of reporter constructs revealed that the sequence between -201 and +71 of mouse FasL gene is essential for CORT-induced FasL expression. The mutation analysis demonstrated that CORT-induced FasL expression is mediated via an NFAT binding element located in the -201 to +71 region. Co-transfection studies with an NFAT2 expression vector and reporter construct containing -201 to +71 region of FasL gene showed that NFAT2 confer a strong inducible activity to the FasL promoter at its regulatory region. In addition, chromatin immunoprecipitation assay further confirmed the results of reporter gene studies by showing the specific binding of NFAT2 to the -201 to +71 region.

**Conclusion:**

In the present study, we demonstrated that NFAT2 directly stimulates transcription of FasL in high level CORT-treated mLTC-1. In conclusion, the present study provides further evidence for our finding that CORT-induced FasL expression in Leydig cells is mediated by NFAT.

## Background

Fas and its ligand FasL are a pair of trans-membrane proteins critically involved in apoptosis. Fas is expressed in many types of tissues, whereas FasL expression is more restricted [1]. FasL expression is predominantly found in activated T cells. However, its expression is also reported in and outside of the immune system, such as NK cells, macrophages and Sertoli cells in testis [2-6].

In male mammalian, the sexual hormone testosterone is secreted by Leydig cells in testis. We have recently demonstrated that the high levels of corticosterone (CORT, glucocorticoid in rat) that are typically achieved during stress induce apoptotic death of Leydig cells and the activation of the Fas/FasL system, cleavage of procaspase-3, loss of mitochondrial membrane potential and increased ROS generation are all implicated in the process of CORT-induced Leydig cell death [7-9]. But the mechanism of FasL expression regulation in CORT-mediated Leydig cell apoptosis is unclear. Transcriptional regulation of FasL expression is a complicated process, which could be involved in a variety of transcriptional factors [10]. It was known that FasL expression is dependent on nuclear factor of activated T-cells (NFAT) in T lymphocyte. In our another study, it was found that the expression of FasL was increased in 100 nM CORT-treated rat Leydig cells and the increased expression could be alleviated by NFAT inhibitor CsA. Meanwhile, Western blot analysis of NFAT protein in the cytoplasm and nuclei demonstrated that NFAT was activated by CORT treatment [11]. These results indicated that NFAT is involved in CORT-induced FasL expression in Leydig cells, but the molecular basis for this process is not clear yet. Therefore, the present study is dedicated to investigate how NFAT be involved in regulation of FasL transcription.

## Results

### Analysis of NFAT2 expression in Leydig cells

**Figure 1:**
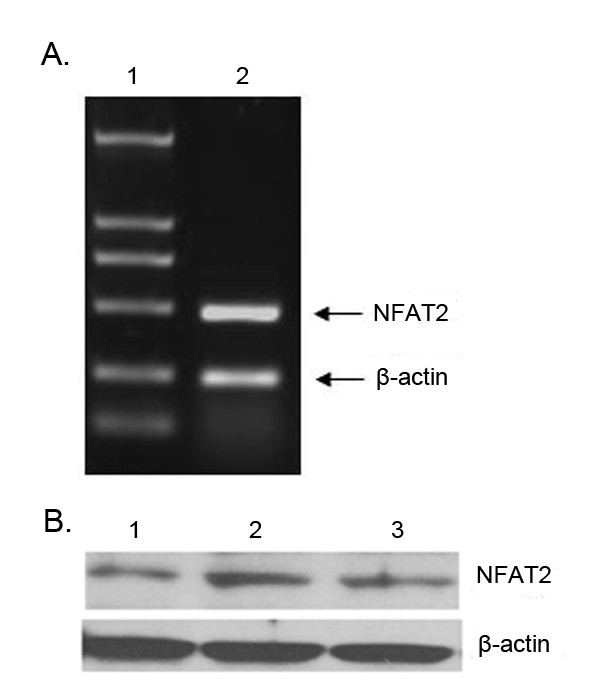
**Expression of NFAT2 in Leydig cells**. A. NFAT2 was determined by RT-PCR using specific primers. β-actin was used as a control to monitor RNA quality. Lane 1 is the marker. The DNA was visualized under UV immediately after staining with ethidium bromide. B. Western blotting was used to determine the expression of NFAT2 protein in Leydig cells. Lane 1. The lysates from primary rat Leydig cells; Lane 2. The lysates extracted from T cells as a positive control; Lane 3. The lysates from mLTC-1 cells. β-actin was used as a control to monitor protein quality. The proteins were resolved by SDS-PAGE and immunoblotted with anti-NFAT2. Strong bands of NFAT2 protein in mLTC-1 and primary rat Leydig cells can be seen, which is similar to that in T cells.

To observe whether NFAT is expressed in Leydig cells, NFAT mRNA and protein expression in Leydig cells was assessed using RT-PCR and Western Blotting respectively. As shown in Fig. 1A, a band corresponding to NFAT mRNA was observed. Fig 1.B shows that a 100 kDa band corresponding to NFAT was identified by anti-NFAT antibody.

### Assay of FasL expression in mLTC-1 cells

**Figure 2:**
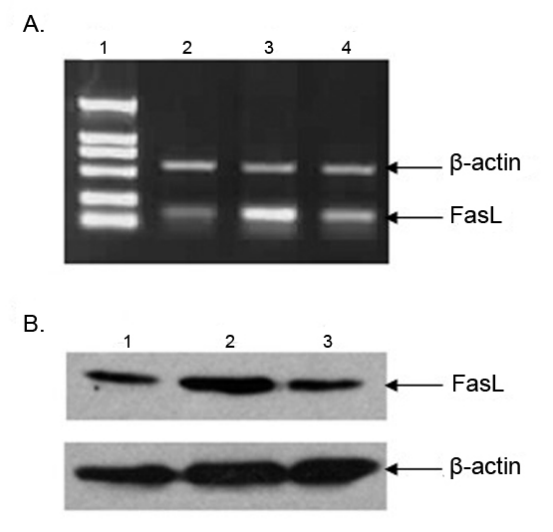
**The enhancement of FasL mRNA and protein expression in CORT-treated mLTC-1 cells**. A. Expression of FasL mRNA was determined by RT-PCR in mLTC-1 cells. Band intensity was normalized to the β-actin product generated using specific primers to β-actin. Lane 1. Marker; Lane 2. The level of FasL mRNA in mLTC-1 cells untreated with CORT; Lane 3. The level of FasL mRNA in mLTC-1 cells treated with 100 nM CORT for 12 h; Lane 4. The level of FasL mRNA in mLTC-1 cells treated with 100 nM CORT plus 100 ng/ml CsA for 12 h. The level of FasL mRNA was increased following the CORT treatment. The mRNA level was decreased in mLTC-1 cells with CORT and CsA treatment. B. Expression of FasL protein in mLTC-1 cells was determined by Western blot. Lane 1, 2, 3 is without CORT treatment, with CORT treatment and CORT plus CsA, respectively. The expression of FasL was increased with CORT treatment and decreased with CORT plus CsA treatment.

For assay of FasL mRNA in mLTC-1 cells, a total RNA were extracted from the cells. A signigicantly increased level of FasL mRNA was found in mLTC-1 cells treated with CORT (Fig. 2.A). To further assay the product of FasL expression in mLTC-1 cells subjected to 100 nM CORT treatment, the lysates from mLTC-1 cells were examined by Western blotting. As predicted, immunoblotting with anti-FasL antibody revealed the expression of a band with a M.W about 40 kDa (Fig 2.B). Taken together, these results indicate that CORT increase expression of FasL in mLTC-1 cells. To analyze the effect of CsA in CORT-induced FasL expression. mLTC-1 cells were treated with 100 nM CORT and 100 ng/ml CsA for 12 h followed by detection of FasL mRNA with RT-PCR and FasL protein with immunoblotting. As shown in Fig. 2A, B, the addition of CsA alleviated CORT-induced up-regulation of FasL expression.

### RNA interference-mediated silencing of NFAT2 gene

**Figure 3:**
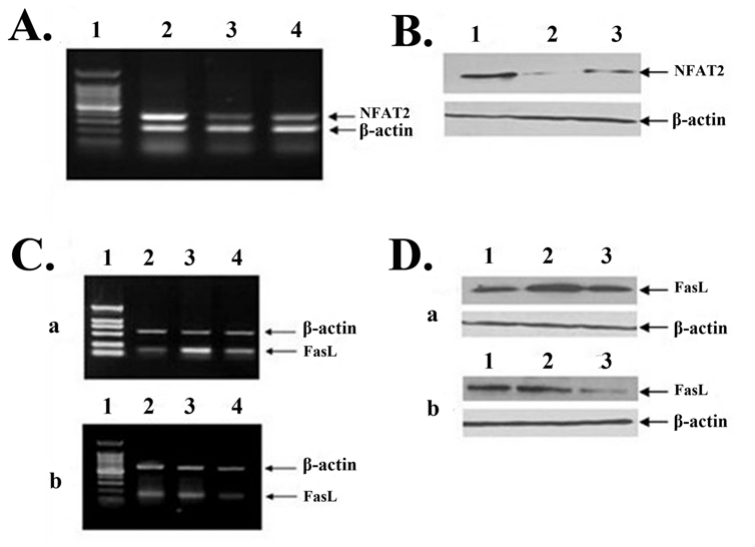
**Suppression of NFAT2 expression by siRNA attenuates CORT-induced expression of FasL**. A. After cells were transfected with the siRNA, NFAT2 siRNA394 or NFAT2 siRNA1232, targeting NFAT2 gene for 48 h, the expression of NFAT2 mRNA was analyzed by RT-PCR with primers specific to NFAT2. The cells transfected with scrambled siRNA served as negative control, and beta-actin mRNA served as internal controls. Lane1. 100-bp marker; Lane 2. Scrambled siRNA; Lane3. NFAT2 siRNA394; Lane 4. NFAT2 siRNA1232. B. Cells were transfected with the scrambled siRNA or siRNA targeting NFAT2 gene for 72 h, and expression of NFAT2 protein was analyzed by Western blotting with specific antibody. Lane1. Scrambled siRNA; Lane 2. NFAT2 siRNA394; Lane 3. NFAT2 siRNA1232. C. Expression of FasL mRNA was determined by RT-PCR in wild mLTC-1 (a) and mLTC-1 transfected with NFAT2 siRNA394 (b) after cells were treated with CORT or CORT plus CsA. Lane 1. Marker; Lane 2, 3 and 4 is without CORT treatment, with CORT treatment and CORT plus CsA treatment, respectively. D. Expression of FasL protein was determined by Western blotting in wild mLTC-1 (a) and mLTC-1 transfected with NFAT2 siRNA394 (b) after cells were treated with CORT or CORT plus CsA. Lane 1, 2 and 3 is without CORT treatment, with CORT treatment and CORT plus CsA treatment, respectively.

The RT-PCR and Western blotting were used to evaluate the efficiency of siRNA on NFAT2 expression. After transfection of mLTC-1 cells with siRNA targeted to NFAT2, expression of NFAT2 is specifically abrogated compared with the control siRNA (Fig. 3A, B). To determine whether NFAT2 is responsible for FasL expression in mLTC-1 cells, FasL expression were also detected by RT-PCR and Western blotting. As shown in Fig. 3C, D, FasL expression is decreased in siRNA-transfected mLTC-1 cells, whereas control siRNA-transfected cells were not affected.

### CORT-induced NFAT2-GFP nuclear translocation

**Figure 4:**
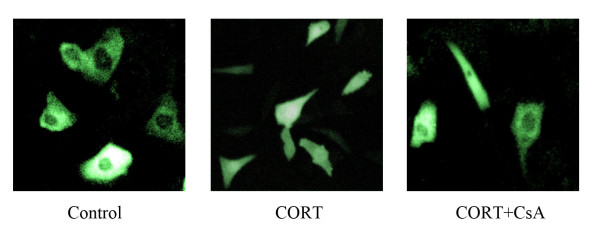
**CORT-induced nuclear translocation of NFAT2 in mLTC-1 cells**. mLTC-1 cells were transiently transfected with NFAT2-GFP expressing vector. 36 h after transfection, cells were treated with 100 nM CORT or 100 nM CORT plus 100 ng/ml CsA for 12 h. The cells were rinsed with PBS and fixed. Representative fields of cells were viewed by Confocal microscopy and photographed.

To determine whether CORT induces nuclear translocation of NFAT2, plasmids expressing a NFAT2-GFP fusion protein were transfected into mLTC-1 followed by CORT or CORT plus CsA treatment. As shown in Fig. 4, NFAT2-GFP was distributed evenly throughout the cytoplasm in Control cells. After CORT treatment, NFAT2-GFP translocate to the nucleus, which was blocked by NFAT inhibitor CsA. These results suggest that high level of CORT induces nuclear translocation of NFAT in mLTC.

### Enhancement of FasL promoter activity by CORT treatment

**Figure 5:**
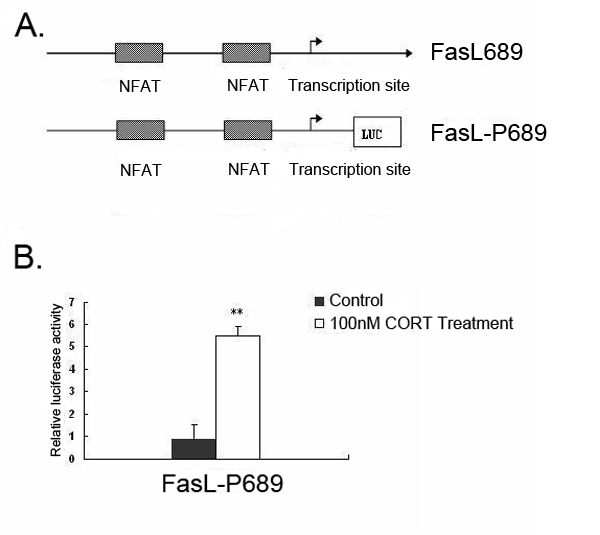
**The enhancement of transcriptional activity of FasL promoter in CORT- treated mLTC-1 cells**. A. Schematic representations of known transcription factor binding sites in the 5' regulatory region of the FasL gene and reporter-luciferase constructs used in these studies. Lengths of the promoter regions in the 5' truncation constructs are relative to the transcription start site. B. Analysis of transcriptional activity of FasL promoter in mLTC-1 cells subjected to CORT treatment. mLTC-1 cells were transiently transfected with luciferase reporter constructs containing the -618 to +71 bp region of FasL promoter (FasL-P689). The transfected cells were treated with vehicle as control or treated with 100 nM CORT for 12 h. The luciferase activity, normalized to an internal *Renilla *control, was compared with the activity of the transfected but no treated cells. There data are representative of at least three independent experiments. ** denotes a significant difference compared with control at *P *< 0.01.

To determine whether CORT regulates FasL promoter activity, FasL-P689 reporter construct was used to transiently transfect mLTC-1 cells. Fig. 5A shows schematically the -618 to +71 bp 5' regulatory region of FasL promoter (FasL689) and the -618 to +71 bp FasL luciferase construct (FasL-P689). As shown in Fig. 5B, CORT induced a 6-fold increase in luciferase activity over untreated cells. This result suggests that CORT treatment enhanced FasL transcriptional activity in mLTC-1 cells. These data show that the -618- +71 region is sufficient for CORT-mediated activation of FasL expression.

### Transfection of mLTC-1 cells with deletion constructs of FasL promoter

**Figure 6:**
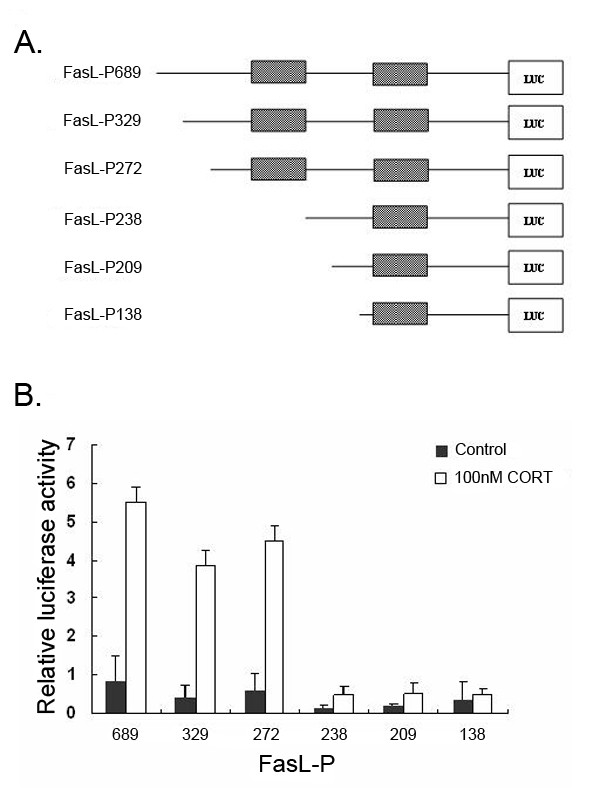
**Delineation of CORT-responsive *cis*-acting elements in the FasL promoter**. A. Schematic representation of FasL 5' truncation reporter- luciferase constructs. B. A series of FasL-luciferase reporter constructs were transfected into mLTC-1 cells. After overnight incubation, the mLTC-1 cells were treated with 100 nM CORT for an other 12 h. The cells were lysed and assayed for luciferase activity. The luciferase activity was compared with the normalized activity of the transfected but not treated cells. These data are representative of at least three independent experiments and are normalized to an internal *Renilla *control. ** denote a significant difference compared with control at *P *< 0.01. * denote a significant difference compared with control at *P *< 0.05.

To localize the promoter regions responsive to CORT-induced FasL expression and define the minimal functional region, a detailed 5' deletion analysis of -618 to +71 bp region was undertaken with FasL-P689, FasL-P329, FasL-P272, FasL-P238, FasL-P209, FasL-P138. These constructs were transiently transfected into mLTC-1 cells followed by 100 nM CORT treatment. As shown in Fig. 6, a significantly increased luciferase activity was presence in FasL-P689, FasL-P329 and FasL-P272. FasL-P272 showed the maximal promoter activity and minimal 5' regulation region among these constructs. These data suggest that the 272 bp sequence might be the regulatory elements that respond to CORT-induced signaling and served as a basis for mutational analysis.

### Transfection of mLTC-1 cells with mutant FasL promoter constructs at 272 bp sequence

**Figure 7:**
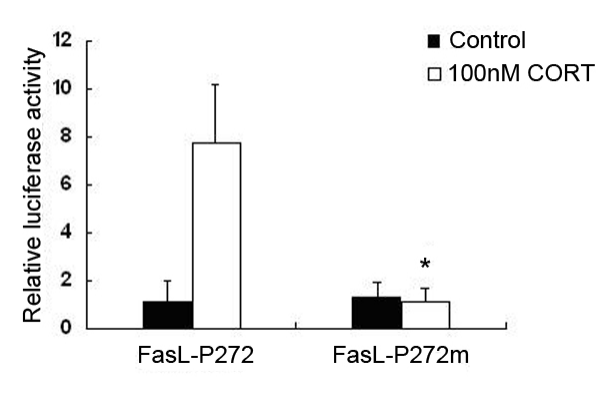
**Transient transfection of mLTC-1 cells with mutant construct of the FasL promoter**. Cultured mLTC-1 cells transfected with FasL-P272 or FasL-P272m were treated with 100 nM CORT for 12 h followed by luciferase activity assays. The transfected cells which were treated with vehicle served as controls. The mLTC-1 cells with CORT treatment dramatically increased transcriptional activity of FasL-P272, but not FasL-P272m. Up to 80% of the FasL promoter activity was lost in mLTC-1 cells transfected with the FasL-P272m when the cells were incubated with 100 nM CORT, compared with that in the cells transfected with FasL-P272. Asterisk indicates that the difference between FasL-P272 and FasL-P272m is statistically significant (*P *< 0.05).

Figure 6 shows that there are two NFAT site in the 272 bp sequence. The transcription activity of FasL-P272 is several times higher than that of FasL-P238. We infer that the distal NFAT site is more important than the proximal NFAT site. To further determine the function of NFAT at 272 bp sequence, the mutant FasL-P272 constructs (FasL-P272m), in which a mutation was introduced at the distal NFAT site, was used in transient tranfection assays. It was observed that up to 80% of luciferase activity indicating FasL promoter activity was lost after the mutant construct was transiently transfected into mLTC-1 cells followed by 100 nM CORT treatment. mLTC-1 cells were transfected with the FasL-P272m (Fig. 7). This observation indicates that the GGCGGAAA sequence (NFAT binding site) is the critical site for FasL promoter activity in CORT-treated mLTC-1 cells.

### Activation of FasL promoter activity with transfection of NFAT expression construct

**Figure 8:**
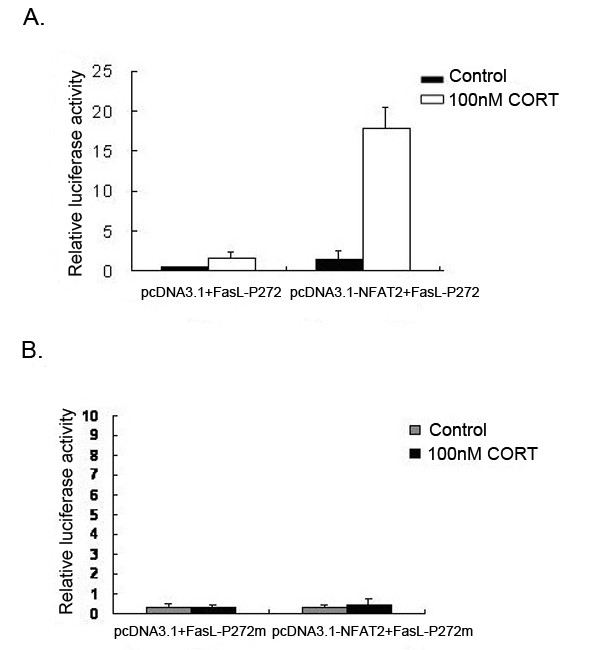
**Co-transfection of NFAT expression vector activates FasL promoter**. A. Leydig cells were co-transfected with FasL-P272 (0.4 μg) and pcDNA3.1-NFAT (NFAT expression vector, 0.8 μg), or empty pcDNA3.1. Luciferase activity was determined after 48 h incubation with CORT. CORT treatment significantly increased the luciferase activity compared with controls. The increase of Luciferase activity in the cells co-transfected with pcDNA3.1-NFAT and FasL-P272 is several times higher than that in the cells cotransfected with pcDNA3.1 and FasL-P272. B. Leydig cells were co-transfected with FasL-P272m (0.4 μg) and pcDNA3.1-NFAT (0.8 μg), or empty pcDNA3.1. Luciferase activity was determined after 48 h incubation with CORT. CORT treatment did not induce elevation of luciferase activity in cells either transfected with or without pcDNA3.1-NFAT. Results represent the average of three independent transfection experiments.

To further establish that whether the NFAT directly activates FasL promoter, cotransfection with the NFAT expression vector pcDNA3.1-NFAT and FasL-P272 were conducted in mLTC-1 cells. pcDNA3.1-NFAT is under the control of pcDNA3.1. pcDNA3.1-NFAT with FasL-P272 induced potent promoter activity. However, co-transfection of pcDNA3.1-NFAT with FasL-P272m induced little promoter activity (Fig. 8). The data indicate that NFAT is capable of binding to the GGAAA site of the FasL-P272 promoter construct.

### Specific Binding of NFAT2 to the FasL promoter in vivo

**Figure 9:**
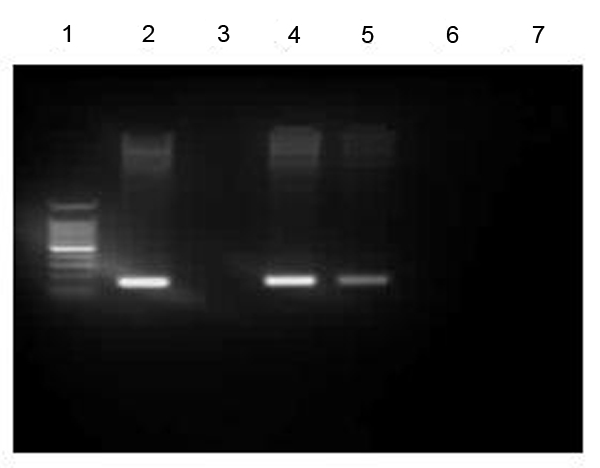
**Recruitment of NFAT to the FasL promoter region**. ChIP analysis of the FasL promoter region was carried out on cells treated with CORT. The chromatin DNA selected by anti-NFAT antibody was amplified with FasL specific primers. Lane 1, marker; Lane 2, the input of mLTC-1 cells; Lane 3, the ChIP DNA from mLTC-1 cells untreated with CORT. Lane 4, the ChIP DNA from mLTC-1 cells treated with 100 nM CORT; Lane 5, the ChIP DNA from mLTC-1 cells treated with 100 nM CORT and 100 mg/ml CsA; Lane 6, non-specific IgG; Lane7, non-specific primer.

Chromatin immunoprecipitation (ChIP) assay was employed to assess the specific binding of NFAT to the target site in promoter region of the FasL gene in vivo (Fig. 9). A five-fold enrichment for FasL promoter DNA over DNA immunoprecipitated with a nonspecific immunoglobulin G (IgG) control antibody was demonstrated. This result indicates that NFAT bind directly to the FasL promoter region.

### Detection of CORT-induced apoptosis of mLTC-1 cells

**Figure 10:**
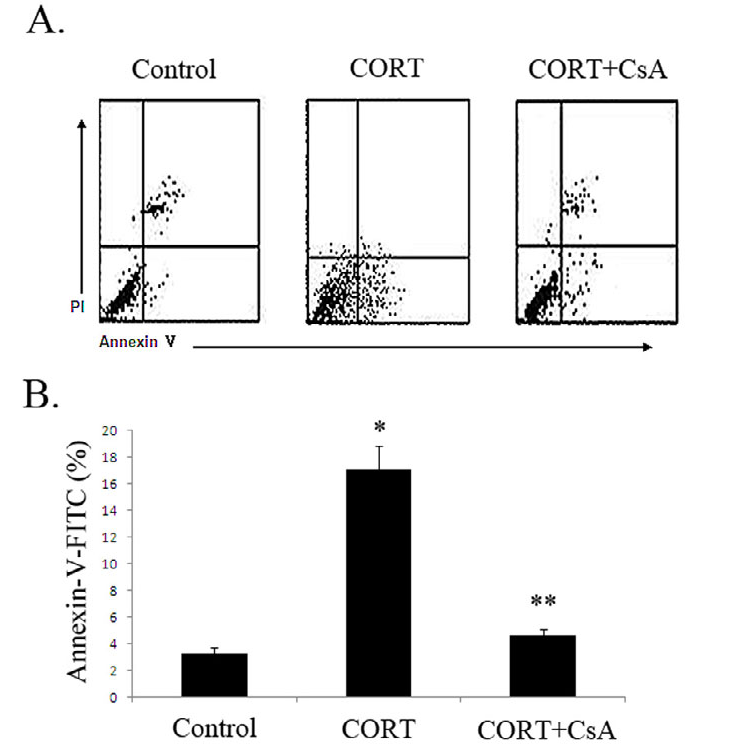
**Detection of CORT-induced apoptosis of mLTC-1 cells**. A. mLTC-1 cells were incubated with either 100 nM CORT or 100 ng/mL CsA plus 100 nM CORT. The Leydig cells treated with a vehicle (Dimethyl Sulfoxide, DMSO) served as control. After 12-h incubation, the mLTC-1 cells were analyzed by FACS with annexin-V/PI double staining. The cells in the right lower quadrant were designated apoptotic (propidium iodide (PI)-negative/annexin V-fluorescein isothiocyanate-positive), the cells in the left lower quadrant were designated alive (PI-negative/annexin V-FITC-negative), the cells in right upper quadrant were designated dead (PIpositive/annexin V-FITC-positive), and the cells in left upper quadrant were designated damaged (PI-positive/annexin V-FITC-negative). B. The mLTC-1 cells treated with CORT showed increased frequencies of apoptotic labeling compared with the control. The Leydig cells treated with CORT plus CsA showed decreased frequencies of apoptotic labeling compared with the cells treated with CORT alone. * denote a significant difference compared with control at P < 0.05. ** denote a significant difference compared with cells treated with CORT at P < 0.05.

To verify whether 100 nM CORT which induces up-regulation of FasL in mLTC-1 could induce apoptosis, PI and Annexin V-labeled FACS assay were performed (Fig. 10). CORT treatment resulted in a significant increase of apoptotic frequency of mLTC-1 cells, and CsA, the specific inhibitor of NFAT, alleviated the increase of apoptosis.

## Discussion

The receptor-ligand pair of Fas and FasL mediates apoptosis in a wide variety of different cell populations. The importance of these interactions has been convincingly demonstrated in murine and humans possessing loss of function mutations in either molecule [15-18]. The biological importance of FasL has been highlighted recently by work demonstrating its critical role in both activation-induced cell death in T cells and in the maintenance of immune privilege within certain tissues [19-21]. However, relatively little is known about factors that regulate inducible expression of FasL outside of the immune system. Our previous studies established a critical role for FasL in Leydig cell via its action in CORT-induced apoptosis. We are the first to demonstrate that involvement of NFAT on regulation of FasL expression in CORT-induced Leydig cell apoptosis [11]. But the molecular mechanisms of NFAT-mediated up-regulation of FasL expression in CORT-induced Leydig cell apoptosis are unknown. Regulation of FasL expression is a complicated process, which could be involved in a variety of trascriptional factors including NFAT, SP1, early growth response gene (Egr) and so on. The NFAT family of transcription factors is composed of four family members NFAT1, NFAT2, NFAT3 and NFAT4, and is best known for its role in the regulation of the T cell immune response [22-27]. The principal function of NFAT in T cells is to couple stimulation of the T cell antigen receptor to changes in the expression of a number of cytokine and other immunologically important genes. NFAT are regulated primarily at the level of their subcellular localization through the actions of serine/threonine phosphatase calcineurin (CaN). In resting cells, NFAT family members are normally located in the cytoplasm in a hyperphosphorylated latent form. However, following activated CaN directly dephosphorylates NFAT proteins, nuclear import and increased intrinsic DNA binding activity occur, which could be specially inhibited by CsA. Once located in the nucleus, NFAT are then free to bind to their target promoter elements and activate the transcription of specific NFAT target genes, either alone or in combination with other nuclear partners.

This aspect of NFAT regulation is likely to be significant, since both the extent and duration of NFAT activity have recently been shown to influence the qualitative pattern of NFAT-dependent gene expression induced during T cell activation [28]. The NFAT signaling pathway is certainly best known for its role in the regulation of the immune response. The NFAT is expressed in many cell types and contributes to diverse cellular functions [22,29-34]. Therefore, it has become increasingly apparent that this pathway also plays an important role in the regulation of a wide variety of cellular responses in a number of other tissues.

Our recent study had verified that NFAT was involved in CORT-induced expression of FasL in rat Leydig cells [11]. Owing to relatively little is known about NFAT regulation of FasL expression outside of the immune system, no doubt, it is very interesting to observe whether NFAT regulate FasL expression through affection FasL promotor activation in CORT-treated Leydig cells.

In the present study, mLTC-1 were selected as cell model due to its suitability for transfection. Although it is widely accepted that mLTC-1 is a favorable model for study on biology of Leydig cells, we validated firstly that CORT-induced up-regulation of FasL expression is also present in mLTC-1 by RT-PCR and Western blotting analysis. Western blotting analysis showed NFAT2 is the predominant isoform in mLTC. However, other members of NFAT family, i. e. NFAT1, NFAT3 and NFAT4 exprssion is very low compared to NFAT2 in mLTC-1 (our unpublished data). It was also shown that NFAT2-GFP chimeric protein translocated from cytoplasm to nuclus after CORT treatment and siRNA-mediated silencing of NFAT decreased the extent of CORT-induced FasL expression. These results were in accord with our finding in rat Leydig cells, i.e. high level CORT activates Fas/FasL signal pathway through activation of NFAT.

Previous studies from other investigators suggested the presence of multiple elements at -689 bp at 5' untranslated region of FasL promoter, which is capable of enhancing transcriptional activity of the FasL promoter in T cells [35]. Furthermore, we predicated the transcription start site and transcription factor binding sites on FasL promoter by analyzing the sequence of FasL 5' untranslated region through online-based computer program. Based on these messages, the -689 bp at 5' untranslated region of FasL promoter was selected as an object to observe NFAT regulation of FasL promoter activity in present study. In order to elucidate the mechanism of CORT-triggered up-regulation of FasL expression in CORT-treated Leydig cells, we cloned -689 bp (-618- +71) at 5' untranslated region of FasL promoter and found that the -618- +71 region is available and sufficient for further establishment of mechanisms of NFAT-triggered FasL expression in CORT-treated Leydig cells.

The data presented in this paper firstly suggested that activity of FasL promoter (at -689 bp sequences) was enhanced in CORT-treated mLTC. To establish the minimal function region on FasL promoter which is interaction with NFAT, we synthesized a series of 5' deletion constructs and tested their transcriptional activities in response to CORT treatment. It was firstly found the presence of a high active site situated between -201- +71 (Fig. 8). Our present data showed that there are two NFAT binding sites between -201-+71 region (with total 272 bp) and only one NFAT binding site between -167-+71 region (with total 238 bp). The 272 bp showed the maximal activation in whole FasL promotor sequences. Latinis et al. reported that the binding of NFAT to the GGAAA site on FasL active core sequence is critical for triggering FasL promoter activity [36]. So this specific short sequence was selected as mutated site in present experiment. It was found that the mutated sequence significantly resulted in an inhibition of FasL promoter activity following the nucleotides sequence was mutated. Finally, co-transfection experiments with the NFAT expression vector and FasL promoter with report gene further showed that NFAT enhanced transcriptional activity of FasL promoter by interaction with its -272 bp DNA sequence, thereby regulating FasL promoter activity in CORT-treated Leydig cells. In addition, mutation at -272 bp DNA sequence also resulted in an inhibition of transcriptional activity of FasL promoter in co-transfection experiments. Furthermore, the ChIP result further confirms that NFAT directly bind to FasL promoter sequence in vivo.

## Conclusion

Our present findings provide further insight into the mechanisms involved on the regulation of the FasL expression in response to CORT treatment in Leydig cells. In summary, the mechanisms of transcriptional regulation of FasL expression in CORT-treated mLTC-1 is an activation on transcription factor NFAT. The active transcription factor interact with specific NFAT target genes in FasL promoter sequence at -201 to +71 region, thereby regulating FasL expression.

## Methods

### Cells and reagents

Cell line mLTC-1 (mouse Leydig tumor cell) was purchased from American Type Culture Collection (Cat. No. CRL-2065). CORT was purchased from Sigma Chemical Co. QuickChange Site-Directed Mutagenesis Kit was purchased from Stratagene Inc (La Jolla. CA, Cat. No.200518). Qiagen Plasmid Maxiprep Kit was obtained from Qiagen (Santa Clarita, CA, Cat. No.12162) The Dual-Luciferase Reporter Assay System was purchased from Promega Co (Madson, WI, Cat. No.E1910). The Fluo-3/AM was purchased from Sigma Co. Specific antibody for FasL was obtained from Oncogene (Cambridge, MA, Cat. No. PC78). The anti-NFAT2 was obstained from Santa Cruz Biotechnology, Inc (Santa Cruz, CA, Cat. No. SC-1149).

### Isolation of primary rat Leydig cells

Adult Leydig cells were isolated from the 90-day-old rats according to the procedure of Sriraman et al. [12], which is a modification of the procedure described by Klinefelter et al. [13]. The decapsulated testes were subjected to collagenase digestion in a 50 mL plastic tube containing 10 mL medium with collagenase (600 units) and DNase (750 units). The tubes were placed in a shaking water bath and constantly agitated (50 times/min) at 34°C for 15–20 min until the seminiferous tubules were separated. The enzyme action was terminated by adding excess medium. The tubules were allowed to settle by gravity and the medium, consisting of interstitial cells, was aspirated and filtered through a 100 μm nylon mesh. The filtrate was centrifuged at 250 × g for 10 min at 25°C, which yielded a crude interstitial pellet. The pellet obtained was suspended in 35 mL 55% isotonic Percoll with 750 units DNase in Oakridge tubes. The tubes were centrifuged at 20 000 × g for 1 h at 4°C. Percoll fractions corresponding to densities of 1.070–1.090 g/mL were collected and the cells present in this fraction were pelleted down by centrifugation at 250 × g for 10 min at 25°C after diluting it with 3–4 volumes of the medium. The purities of the isolated cell fractions were evaluated by histochemical staining for 3β-hydroxysteroid dehydrogenase activity, with 0.4 nm etiocholanolone as the steroid substrate [14]. The enrichment of the Leydig cells was up to a purity of 85% on average.

### RNA isolation and RT-PCR

Total RNA was prepared from mLTC-1 cells using the Trizol Reagent (Gibco, Cat. No.15596-026) according to the manufacturer's instruction. First strand cDNA synthesis was performed using Superscript II reverse transcriptase (Invitrogen, Carlsbad, CA, Cat. No. A3500) in a reaction using 2 μg of total RNA primed with random hexamers in a total reaction volume of 20 μl. Following first strand synthesis, 10% of the reaction volume was used as a DNA template for amplification by polymerase chain reaction (PCR). For detection of NFAT2, the forward and reverse primers were 5'-GCCCTGACCACCGATAGCAC-3' and 5'-GCTGCCTTCCGTCTCATAGTG-3', respectively. PCR was performed for 30–32 cycles with denaturing at 94°C for 1 min, annealing at 58°C for 1 min and extension at 72°C for 1 min, in a GeneAmp PCR System 9600 (PerkinElmer, Norwalk, CT, USA). For detection of FasL, specific oligonucleotide primers were used as follows: forward primers; 5'-ATTGGCACCATCTTTACT-3' and reverse primers; 5'-CCTTAGAATCTGTTTGTCC-3'). PCR was performed for 28–30 cycles with denaturing at 95°C for 50s, annealing at 58°C for 45s and extension at 72°C for 50s. The signal intensities of the amplified fragments of NFAT2 and FasL were normalized to β-actin using a densitometer. Amplified PCR products were separated by agarose gel electrophoresis and detected by ethidium bromide staining. For amplification of NFAT2, the forward and reverse primers were 5'- CCG GAA TTC GAC ATG ACG GGG CTG GAG CAG GAC CCG GAG -3'and 5'- CGC GGA TCC TCG TAA ATA AAA CAC CCT AGA AAG TTG AAA-3', respectively. PCR was performed for 34 cycles with denaturing at 94°C for 50s, annealing at 60°C for 45s and extension at 72°C for 120s. The NFAT2 cDNA was purified by QIAquick PCR Purification Kit.

### Western blot analysis

mLTC-1 and primary rat Leydig cells were lysed in Ripa buffer (1% NP-40, 0.1% SDS, 0.5% DOC, 150 mM NaCl, 10 mM Tris-HCl, and PMSF mixture) at 4°C for 30 min. Protein concentrations were determined by the Bradford dye-binding assay using bovine serum albumin as a standard. Aliquots of cell extracts containing equal amounts of protein were separated by SDS-polyacrylamide gel electrophoresis on 8% gels using the Laemmli buffering system, then proteins were transferred to Immuno-Blot polyvinylidene difluoride membranes (Bio-Rad, Hercules, CA) and blocked by rocking for 1 h at room temperature in blocking buffer (Tris-buffered saline with 0.1% Tween 20 and 5% nonfat dry milk). Blots were exposed to primary antibodies for 1 h at room temperature, multiply washed with Tris-buffered saline with 0.1% Tween 20 (TBST), treated with secondary antibody (HRP-conjugated) for 1 h, and followed by a final series of washed with TBST. Primary (goat polyclonal anti-NFAT2 or rabbit polyclonal anti-FasL) and secondary antibodies were prepared in blocking buffer. Signals were detected with an Enhanced Chemiluminescence kit (ECL Pierce, Rockford, IL) and analyzed using Adobe Photoshop 6.0 software.

### RNA interference

The following sequences specific for mouse NFAT2 gene were used: NFAT2 siRNA394 (Sense: 5'-GGA GGU GGA AGA CGU ACU UTT-3'; Antisense: 5'-AAG UAC GUC UUC CAC CUC CTT-3') and NFAT2 siRNA1232 (Sense: 5'-CCG UCA CAU UCU GGU CCA UTT-3'; Antisense: AUG GAC CAG AAU GUG ACG GTT-3'). The Lipofectamine 2000 was used to deliver siRNA (100 pmol) into cultured mLTC-1 cells (5 × 104) in 6-well plates according to the manufacturer's protocol. The double-strand scrambled siRNA (5'-UUC UCC GAA CGU GUC ACG UTT-3') were used as negative control. The cultures were incubated for six hours and then washed and incubated in fresh medium. All siRNA were obtained from Shanghai Genepharma Co. Inc. (Shanghai, China).

### Plasmid constructions

Genomic DNA was isolated from C57BL6 using DNAeasy kit (Qiagen, Valencia, CA, Cat. No.69504). A 689 bp PCR fragment corresponding to the promoter region of FasL (-618- +71) was generated using the upstream primer 5'-CGGGGTACCGTACCTCAGTTTTCATCTGGTGACCAGAAG-3' and the downstream primer 5'-CCCAAGCTTGCACCCAGCCCCAGGAAA GG-3' in a PCR using platinum Pfx high fidelity polymerase (Invitrogen, Cat.No. 11708013) and 50 ng of genomic DNA template. The resultant 689 bp fragment was gel-purified, and cloned into pGEM ^®^-T Easy vector (Promega, Cat.No. A1360) and sequenced for orientation and fidelity. This plasmid (pGEM-689pCD95L) was served as the source of the insert for further plasmid constructions. To make the -618 to +71 luciferase reporter plasmid, the 689 bp insert was excised with *Kpn I *and *Hind III *and ligated into the *Kpn I/Hind III *site of pGL3 Basic luciferase vector (Promega, Cat.No.E1751). To make the 5'truncation mutants, shorter fragments were generated using new upstream primers, and the common downstream primer was used to make the -618 to +71 insert. For the -258 to +71 fragment, the upstream primer 5'-CGGGGTACCCAGGCAAGCCTGGTTTACCAGC C-3' and the common downstream were used. To generate the -201 to +71 fragment, the upstream primer 5'-CGGGGTACCCGAAGACTTGTCGTCAGAA ATTTC-3' was used. The -167 to +71 fragment was produced using 5'-CGGGGTACCCTTCCTGGGGTT GCTGTGAGC- 3'. The -138 to +71 fragment was produced using 5'-CGGGGTACCGCTTCTCAGCTTC A ATGCAAG-3'. The -67 to +71 fragment was produced by using the upstream primer 5' -CCCAAGCTT GCACCCAGCCCCAGGAAAGG-3'. The resultant fragment were subcloned into the polylinker site of pGEM-T vector and sequenced for orientation and fidelity. Luciferase reporter constructs were produced by excising the various fragments with *Kpn I *and *Hind III *and then subcloning the fragments into the polylinker site of pGL3-basic.

NFAT2 cDNA sequences were digested with EcoRI and BamHI, and ligated into the EcoRI/BamHI site of pEGFP-C1 plasmids to construct the NFAT2-GFP fusion protein expression vector.

### Site-directed mutagenesis

Mutant construct was generated by using the QuickChange Site-Directed Mutagenesis Kit. The mutation was verified by DNA sequencing. Mutant of the NFAT site in FasL promoter (FasL-P272m) were synthesized by Sangon Co.: 5'-GTCGTCAGAAATTTCTGGGAATCAACTTCCTGGGGTTGCTGTGAG -3'.

### Transient transfections and luciferase reporter assays

For transfection analysis, luciferase assays were performed according to the manufacturer's instruction using the Lipofectamine™ 2000 reagent (Invitrogen Co. Cat. No.11668-019). Briefly, the cells were seeded into 24-well plates at a density of 5 × 10^5 ^cells/well and incubated at 37°C overnight. Cells were then placed in DMEM with 10 % fetal bovine serum containing 1 μg of luciferase plasmid, 0.1 μg of pRL SV40 reporter plasmid, and 2 μg of Lipofectamine™ 2000. Thirty-six hours following transfection, the cells were treated with 100 nM CORT. Following the treatment, the Dual Luciferase Assay was performed by lysing the cells in Passive Lysis Buffer, and reading the relative light units of one-fifth the lysate with both the firefly substrate and the renilla substrate using a luminometer (Monolight 2010, Analytical Luminescence Laboratory, SanDiego, CA). Each transfection was performed in triplicate and in a minimum of three independent experiments.

### Confocal microscopy analysis of NFAT2-GFP nuclear translocation

mLTC-1 were transiently transfected with NFAT2-GPF expression vector. At 36 h after transfection, cells were treated with 100 nM CORT, or 100 nM CORT plus 100 ng/ml CsA for 12 h. NFAT2-GFP expression and localization was monitored by Confocal microscopy.

### Chromatin immunoprecipitation assay

The chromatin immunoprecipitation (ChIP) assay was performed according to the manufacturer's protocol. mLTC, cultivated in DMEM containing 10% bovine calf serum, were incubated with 100 nM CORT for 12 h, cross-linked by formaldehyde for 10 minutes at 37°C. The reaction was stopped with 0.125 M glycine. Cells were washed using cold PBS containing protease inhibitors. DNA were sheared by sonicate lysate to lengths between 200–1000 bp. Cell samples were centrifuged for 10 minutes at 13,000 rpm at 4°C and supernatant stored at -80°C as chromatin extracts. For chip analysis, pre-cleared the sample with salmon sperm DNA/protein agarose for 30 min at 4°C. Lysates were subjected to immunoprecipitation with anti-NFAT2 antibodies (Santa Cruz Biotechnology) and non-specific IgG (Santa Cruz) were added individually and incubated at 4°C overnight with rotation. Bound and input chromatin samples were placed in 0.5% (wt/vol) SDS and incubated at 65°C for 4 hours to reverse the formaldehyde cross-linking. DNA was further purified by phenol-chloroform extraction and ethanol precipitated using glycogen as an inert carrier. Of the obtained DNA, 2 μl was used in a 20-μl PCR reaction (*Taq *DNA Polymerase; Qiagen) using the following primers: FasL-F, 5'-CAGTTAGCACAGAGACGCCAAT-3'; FasL-R, 5' -TGCTTCTCTGTGAGACACCCAC-3'.

### Apoptosis assay

mLTC-1 cells were treated with 100 nM CORT or 100 nM CORT plus 100 ng/ml CsA for 12 h followed by detection of apoptosis. The annexin-V was used to assess apoptosis. For the annexin-V assay, the mLTC-1 cells were incubated with 10 μL propidium iodide (PI) and 5 μL FITC-annexin-V (BD Biosciences, Franklin Lakes, NJ, USA) at room temperature for 15 min. Then, the cells were analyzed on FACS. Annexin-V binds to those cells that express phosphotidylserine on the outer layer of the cell membrane, and PI stains the cellular DNA of cells with a compromised cell membrane. This allows for the discrimination between live cells (unstained with either fluorochrome) from apoptotic cells (stained only with annexin-V) and necrotic cells (stained with both annexin-V and PI).

### Statistics

All experiments were performed in triplicate. The Student's t-test was used to determine the statistical significance of the data obtained. *P *< 0.05 or *P *< 0.01 was taken to represent a statistically significant difference between group mean.

## Authors' contributions

W–RC and YC executed experiments in this study. QW performed the cells culture and edited the manuscript. H–BG designed and supervised the study. All authors read and approved the final manuscript.
